# Behavioural and psychological features of PTEN mutations: a systematic review of the literature and meta-analysis of the prevalence of autism spectrum disorder characteristics

**DOI:** 10.1186/s11689-021-09406-w

**Published:** 2022-01-04

**Authors:** Katherine Cummings, Alice Watkins, Chris Jones, Renuka Dias, Alice Welham

**Affiliations:** 1 Department of Neuroscience, Psychology and Behaviour, University of Leicester, Lancaster Road, Leicester, LE1 7HA UK; 2 Neuropsychology Service, Great Ormond Street Hospital, London, WC1N 3JH UK; 3 Department of Psychology, University of Birmingham, Birmingham, B15 2TT UK; 4 Department of Endocrinology and Diabetes, Birmingham Children’s Hospital, Birmingham Women’s, and Children’s NHS Foundation Trust, Steelhouse Lane, Birmingham, UK B4 6NH; 5 Institute of Cancer and Genomic Sciences, College of Medical and Dental Sciences, University of Birmingham, Edgbaston Birmingham, UK B15 2TT

**Keywords:** PTEN, PTEN hamartoma tumour syndrome, Autism spectrum disorder, Development, Cognition, Behaviour, Emotional difficulties

## Abstract

**Background:**

*P*hosphatase and *ten*sin homologue (*PTEN*) is a cancer suppressor gene. Constitutional mutations affecting this gene are associated with several conditions, collectively termed *PTEN* hamartoma tumour syndromes (PHTS). In addition to hamartomas, *PTEN* aberrations have been associated with a range of non-tumoural phenotypes such as macrocephaly, and research indicates possibly increased rates of developmental delay and autism spectrum disorder (ASD) for people with germline mutations affecting *PTEN*.

**Method:**

A systematic review of literature reporting behavioural and psychological variables for people with constitutional *PTEN* mutations/PHTS was conducted using four databases. Following in-depth screening, 25 articles met the inclusion criteria and were used in the review. Fourteen papers reported the proportion of people with *PTEN* mutations/PTHS meeting criteria for or having characteristics of ASD and were thus used in a pooled prevalence meta-analysis.

**Results:**

Meta-analysis using a random effects model estimated pooled prevalence of ASD characteristics at 25% (95% CI 16–33%), although this should be interpreted cautiously due to possible biases in existing literature. Intellectual disability and developmental delay (global, motor and speech and language) were also reported frequently. Emotional difficulties and impaired cognitive functioning in specific domains were noted but assessed/reported less frequently. Methods of assessment of psychological/behavioural factors varied widely (with retrospective examination of medical records common).

**Conclusions:**

Existing research suggests approximately 25% of people with constitutional *PTEN* mutations may meet criteria for or have characteristics of ASD. Studies have also begun to establish a range of possible cognitive impairments in affected individuals, especially when ASD is also reported. However, further large-scale studies are needed to elucidate psychological/behavioural corollaries of this mutation, and how they may relate to physiological/physical characteristics.

**Supplementary Information:**

The online version contains supplementary material available at 10.1186/s11689-021-09406-w.

## Background

*P*hosphatase and *ten*sin homologue (*PTEN*), located on chromosome 10 (10q23.3), was initially reported by Li et al. [1] and governs many processes in the cells which are disrupted in cancer [2]. For this reason, *PTEN* is recognised as a tumour suppressor gene. It has also been shown to play an important role in brain development [3]. *PTEN* mutations are related to an elevated risk of both malignant [4] and benign tumours. Conditions associated with constitutional *PTEN* mutations are collectively known as *PTEN* hamartoma tumour syndromes (PHTS). These include Cowden Syndrome (CS), Bannayan–Riley–Ruvalcaba Syndrome (BRRS) and Lhermitte–Duclos disease [5].

Constitutional *PTEN* mutations are found in 57 to 65% [6–8] and approximately 80% [5] of individuals diagnosed with BRRS and CS respectively. It has been suggested that a distinction between CS and BRRS is unnecessary, with age-related penetrance being the salient difference between features [9]. Indeed, 78% of individuals with a diagnosed constitutional *PTEN* mutation met criteria for both CS and BRRS [10], with common clinical features including (amongst other physical characteristics) hamartomas and macrocephaly [5, 11], the latter of which is reported for 85% of those with a CS diagnosis.

Whilst autism spectrum disorder (ASD) is not a listed criterion for PHTS, it has frequently been reported in patients with constitutional *PTEN* mutations [12, 13]. ASD and *PTEN* were initially linked in 2005 by Butler et al. [14] who reported that three of a group of eighteen individuals with ASD and macrocephaly had germline *PTEN* mutations. Mouse models suggest that deletion of *PTEN* in the cerebral cortex and hippocampus results in increased rates of macrocephaly and abnormal social interactions [15–17].

Whilst idiopathic ASD is considered multifactorial [18], elevated rates of ASD have also been observed in a number of genetic neurodevelopmental syndrome groups, such as Fragile X and Cornelia de Lange syndromes (see [19] for a meta-analysis). Previous research has also indicated that the precise profile of ASD-related behaviours may differ between different genetic syndrome groups and from that seen in idiopathic ASD (e.g. [20, 21]). Furthermore, there is evidence that certain social and emotional characteristics, developmental sequalae and categories of psychological distress may be phenotypic of a number of the more extensively researched genetic neurodevelopmental syndromes. For example, social anxiety may characterise Fragile X syndrome [22], low mood is especially prevalent in Cornelia de Lange syndrome [23], and increased rates of psychosis have been recognised in those with 22q11.2 deletion syndrome. However, in the case of constitutional *PTEN* mutations, behavioural/psychological research remains in its early stages. Whilst a number of papers have now been published in which ASD has been reported in a proportion of study participants with *PTEN* mutations, the overall prevalence of ASD in this population remains unknown. We are not aware of any previous pooled prevalence meta-analyses.

Research into neurodevelopmental, cognitive or behavioural features not related to ASD remains limited for those with *PTEN* mutations. Cognitive dysfunction has most frequently been reported in non-human animals [24–26]. Memory impairments, as well as repetitive and “depression-like” behaviours, have also been reported in *PTEN-*mutated mice [24]. To the authors’ knowledge, no systematic reviews of psychological/behavioural corollaries of constitutional *PTEN* mutations in humans have been published to date. Previous reviews exploring a phenotype for *PTEN* mutations have focused specifically on ASD (without meta-analysing its frequency) [27, 28] or individual disorders and their clinical features [29–31].

### The present review

As is common with newly described conditions, research describing behavioural and psychological differences is often presented in disparate accounts and small studies. The current review aimed to systematically identify and synthesise literature reporting behavioural and psychological characteristics associated with *PTEN* mutations, including ASD, cognitive, emotional, social, sensory and motor aspects. A meta-analysis of prevalence rates of characteristics of ASD was also conducted. This may inform the theoretical understanding of implications of *PTEN* changes, and guide clinical practice and service development for those with *PTEN* mutations and their families.

## Methods

The review was conducted in accordance with the Preferred Reporting Items for Systematic Review and meta-analysis protocols (PRISMA-P) 2015 statement [32].

### Search strategy and selection criteria

Two comprehensive sets of search terms representing, respectively, *PTEN* mutations/PHTS and behavioural/psychological features, were developed (Table 1). These were informed by hand searches of terminology in relevant published research, reference to the OMIM website [33], and consultation with authors in the field and library staff at the Universities of Birmingham and Leicester (UK). These terms were used to search Web of Science, SCOPUS, PsycINFO and Cumulative Index of Nursing and Allied Health Literature between 3rd and 6th February 2020. Filters were applied to ensure the papers were written in English and were in peer-reviewed journal articles from 1997 onwards (when the *PTEN* gene was first reported on).

**Table 1:** Free text search terms of *PTEN* related conditions and behavioural and cognitive characteristics

Search terms	
PTEN	“Pten” OR “pten syndrome” OR “hamartoma syndrome” OR “hamartoma tumour syndrome*” OR “PTEN hamartoma tumour syndrome” OR “pten hamartoma-tumour syndrome” OR “phts” OR “phts syndrome” OR “pten mutation*” OR “pten gene mutation” OR “pten germline mutation*” OR “chromosome 10q23” OR “chromosome 10q23 mutation” OR “chromosome 10q23 deletion*” OR “chromosome 10q23 deletion syndrome” OR cowden OR “cowden syndrome” OR “cowden disease” OR “lhermitte duclos syndrome” OR “lhermitte-duclos syndrome” OR “lhermitte duclos disease” OR “lhermitte-duclos disease” OR “bannayan riley ruvalcaba” OR “bannayan riley ruvalcaba syndrome” OR “bannayan-riley-ruvalcaba” OR “bannayan-riley-ruvalcaba syndrome” OR “proteus like syndrome” OR “proteus-like syndrome” OR “proteus syndrome”
Behavioural and cognitive characteristics	((behavio* OR psych* OR clinical OR emotion* OR cognit* OR mental OR sensory) adj3 (phenotyp* OR abilit* OR disabilit* OR delay OR problem OR difficult* OR disorder* OR impair*)) OR ((mental OR intell* OR learning OR development* OR neurodevelopment*OR motor OR psychomotor OR language OR linguistic OR communicat* OR speech OR verbal) adj3 (abilit* OR disabilit* OR delay OR problem OR difficult* OR disorder* OR impair*)) OR “IQ” OR “mental retardation” OR “autis*” OR “autis* spectrum” OR “asd” OR “autis* disorder*” OR “autis* spectrum disorder” OR sleep OR “sleep disorder” OR “ADHD” OR “attention deficit hyperactiv* disorder” OR “attention deficit disorder” OR “ADD” OR ((attention) adj3 (deficit OR disorder* OR dysfunction)) OR “overactivit*” OR “impulsiv*” OR “mood” OR “depressi*” OR “bipolar” OR “anxi*” OR “obsess*” OR “compulsi*” OR “obsess* compulsi* disorder” OR “ocd” OR ((adaptive OR maladaptive OR challeng* OR aggress* OR self-injur* OR self injur* OR repetiti* OR ritual* OR stereotyp*) adj3 (behavio*)) OR memory OR ((memory) adj3 (impair* OR disorder)) OR “executive function*” OR “problem solving”

Note. Rows were combined using the Boolean operator [AND]

Following the removal of duplicates, 723 titles and abstracts were screened with reference to the following exclusion criteria: (a) no mention of PTEN mutations or diagnosis related to constitutional *PTEN* mutations; (b) non-human or molecular studies; (c) no behavioural, cognitive or developmental aspect, and (d) book chapters. This left 98 articles whose full text was screened using criteria in Table 2.

**Table 2:** Final inclusion and exclusion criteria

Inclusion criteria	Exclusion criteria
Confirmed PHTS or germline PTEN mutation Study reports on behavioural/psychological variables/features Only human participants	Solely biological studies/biomarkers No confirmed PHTS or PTEN mutation Review paper with no novel data Proposal/conference paper Fewer than three participants with confirmed PTEN mutation

**Fig. 1:**
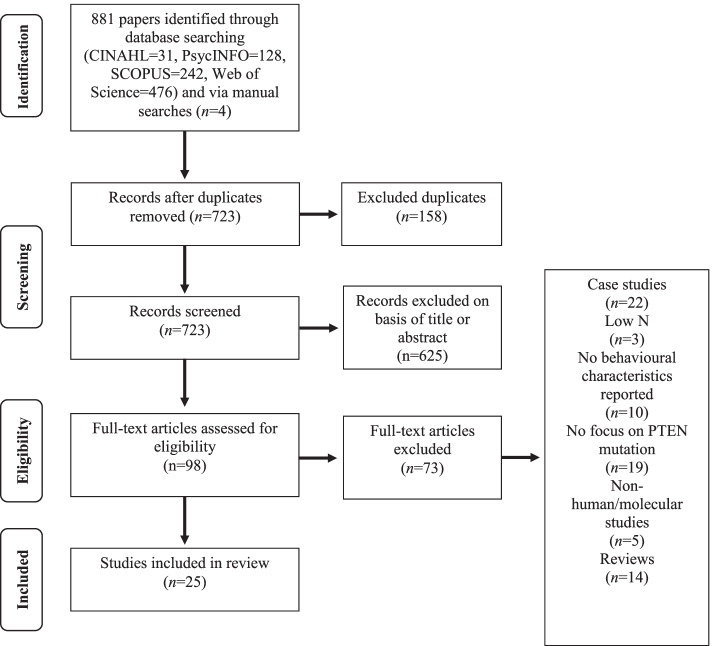
PRISMA flow diagram

Twenty-five articles met full criteria for the review (Fig. 1).

Data regarding sample size, demographic information, recruitment procedure, assessments used, and relevant findings were extracted from included papers.

Two types of studies were identified: those in which participants were recruited/included on the basis of having an identified *PTEN* mutation or *PTEN*-related condition, with behavioural/psychological characteristics examined/reported (group A); those in which participants were selected on the basis of some other factor (e.g. macrocephaly), and these participants were tested for *PTEN* mutations (group B). For group B papers, details of behavioural/psychological characteristics of participants are only reported here for those with *PTEN* mutations or diagnoses of *PTEN*-related conditions.

### Quality/bias appraisal tool

The 25 papers were assessed using the criteria developed by Richards et al. [19], adapted for the current review. Group B studies were rated on sample identification, confirmation of syndrome and quality of assessment of behavioural/psychological characteristic. For papers in group A, an additional criterion was assessed: presence and quality of a comparison group. It should be noted that these criteria are focused on establishing understanding of behavioural/psychological characteristics for this specific group, and should not be taken as ratings of “quality” of the papers more generally.

### Meta-analysis of ASD prevalence

A meta-analysis was conducted of characteristics of ASD prevalence for all papers in which relevant data were reported. To determine the prevalence of diagnosis and characteristics of ASD in those with *PTEN* mutations or PHTS, the total number of these participants reported in the sample, and the number of those described as having ASD, or features of ASD such as “Autistic tendencies”, Asperger’s or “Autistic features” were extracted from each paper. The analysis was also repeated with only papers with more than 10 participants and papers specifically reporting on ASD/autism (excluding those reporting on “tendencies”/“features”).

Meta-analytic weighted prevalence values were generated using the generic inverse variance method. A random effects model was selected to allow for between-study variation reflecting both sampling errors and other factors [34]. Initial Q-Q plots did not indicate marked deviations from normality for the prevalence estimates; therefore, the DerSimonian and Laird method was used to calculate between-studies variance. An additional quality effects model was also employed, with adjusted weightings according to studies’ overall risk-of-bias ratings. In calculating the overall risk-of-bias rating for this analysis, the Assessment criterion focused solely on the assessment of ASD. The “quality of control group” criterion was removed for group A studies.

The existence of possible publication bias was assessed using the visual inspection of a funnel plot, in which the magnitude of the studies’ proportion estimates are plotted against the square roots of the studies’ sampling variances. Following Terrin et al.’s [35] demonstration of the unreliability of subjective judgements of funnel plot symmetry, Egger et al.’s [36] linear regression test of funnel plot asymmetry was also carried out. A trim and fill method was then used to model and correct for asymmetry due to potential publication bias [37, 38], producing adjusted weighted average prevalence estimates.

## Results

### Study characteristics

Information summarising the 25 studies analysed can be found in Tables 3 and 4. Those which were also used in the meta-analysis of prevalence of ASD/characteristic of ASD are marked with an asterisk.

**Table 3:** Summary characteristics of group A articles

Author, year of publication, country of study	Reference Number	Recruitment procedure	Sample size (n)	Comparison Group (no PTEN mutation)	Sex	Age	Assessment tools/methods	Specific details of ASD definition/assessment (for papers used in meta-analysis)	Findings	Quality score
*Balci et al. (2018), Canada	[39]	Patients seen in the Genetics clinic at the Children’s Hospital of Eastern Ontario with PTEN mutations and white matter lesions. No information on referral	11	N/A	10 (M)	4–45 years	Search of medical records	Diagnosis of ASD stated as present or not (no further detail)	Normal development in six participants. Further characteristics: adult onset movement disorder (*n* = 1), bipolar disorder (*n* = 1), memory problems (*n* = 2), GAD (*n* = 2), OCD (*n* = 1), self-harm (*n* = 1), pica (*n* = 1), low processing speed (*n* = 1), speech or language delays (*n* = 3), ADHD (*n* = 2), motor delay (*n* = 3), psychotic episode (*n* = 1) and ASD (*n* = 1)	0.5
*Busa et al. (2015), France	[40]	Children found to carry a PTEN germline mutation between 1 January 2009 to 1 January 2014 with no family history of CS. Identified due to a variety of problems such as lipomas, macrocephaly, facial arteriovenous malformation	7	N/A	3 (M)		Search of clinical data	Diagnosis of ASD stated as present or not (no further detail)	Motor delay (*n* = 3), speech delay (*n* = 4) and ASD reported in one participant	0.5
Busch et al. (2013), USA	[41]	Recruited from an ongoing prospective observational study of PHTS in Cleveland, Ohio between July 2007 and July 2012 and complete 4 h of assessment. Invited if they had undergone mutation analysis or had phenotypic features consistent with CS or BRRS	25 (PTEN = 23, CS = 1, BRRS = 1)	N/A (normative data used in analysis)	7 (M)	5–60 years	Wechsler Adult Intelligence Scale – Third Edition, Wechsler Intelligence Scale for Children – Fourth Edition, or Wechsler Preschool and Primary Scale of Intelligence, WMS-III=Wechsler Memory Scale – Third Edition, Children’s Memory Scale, Trail Making Test, Boston Naming Test, Semantic Fluency, COWA, Wisconsin Card Sorting, Ruff Figural Fluency Test Judgement of Line Orientation, AVLT, Finger Tapping Test and Grooved Pegboard	N/A	Means scores for those with PHTS were significantly lower than controls in motor (fine motor dexterity, large effect), executive functioning (verbal fluency and novel problem solving, medium effect) and memory (immediate and delayed recall, small effect) domains. Global impairments in 12% IQ Range = 80–135, Mean = 107	0.67
Busch et al. (2019), USA	[42]	Recruited from four large tertiary medical centres as part of an ongoing, multicentre prospective study designed to examine the natural history of ASD and germline heterozygous PTEN mutations. All screened by a clinical psychologist to determine if DSM-5 criteria for ASD. No information on referral	PTEN-ASD *n* = 36 and PTEN-no ASD *n* = 23	Macrocephaly-autism *n* = 25	64 (M)	3–21 years	Age appropriate measures of: Global cognitive ability, attention/impulsivity, working memory, processing speed, language, visuo-spatial skills (if severely impaired, inferred from guardians). Guardians completed a number of standardised questionnaires	N/A	PTEN-no ASD not significantly different from control norms on global cognitive measures. Impaired motor and sensory functioning. PTEN-ASD poorer performance than no-ASD in every domain (*d* = 0.41–2.21). Greater behavioural and sensory dysfunction. Severely impaired in verbal and non-verbal IQ, attention, motor and sensory. Moderate impaired on working memory, processing speed, language, visual-spatial and problem behaviour PTEN-ASD and macro-ASD scored similarly in both repetitive behaviour and social responsiveness but lower severity on ADOS-2 which may reflect passivity of PTEN-ASD rather than reduced severity.	0.83
*Ciaccio et al. (2019), Italy	[43]	Participants are paediatric patients seen and diagnosed with *PTEN* mutations in two hospitals in Milan between 2006 and 2017	16	N/A	14 (M)	2 years 5 months–12 years 2 months	Unknown	ASD was assessed in the research centres or in territorial neuropsychiatric using standardised scales (no further detail)	Developmental delay or intellectual disability in 56% of participants. ASD in 25% and normal development in 2 participants	0.58
Frazier et al. (2015), USA	[44]	Unknown	PTEN-ASD *n* = 17	Macrocephaly-ASD *n* = 16, ASD without macrocephaly *n* = 38, healthy controls *n* = 14	67 (M) PTEN: 13 (M)	11.4–14 years (means)	ADI-R, clinical observations, Autism Diagnostic Observation Schedule, Social Responsiveness Scale, Mullen Scales of Early Learning or the Wechsler Abbreviated Scale of Intelligence, Conners’ Continuous Performance Test and Wide Range Assessment of Memory and Learning	N/A	Reduced FSIQ, verbal IQ, non-verbal IQ, in PTEN-ASD group compared to other ASD groups and healthy controls (smallest Wald *Χ*^2^ (3) = 16.86, *p* < .001). Processing speed, working memory, auditory immediate memory and adaptive function (most notably community living) was also reduced in the PTEN-ASD group compared to the macrocephaly-ASD group (Cohen’s *d* = 1.15, 1.07, 0.96, 0.94)	0.67
*Hansen-Kiss et al. (2017), USA	[45]	Retrospective chart review in a paediatric population.“Problem List” on electronic medical records (EPIC) searched for: PTEN mutation, PTEN hamartoma tumour syndrome, CS and/or BRRS. “Laboratory Testing” section was queried for positive/pathogenic results on PTEN gene characterisation gene sequencing	47	N/A	29 (M)	1–26 years	Search of medical records which, for some participants, reported on results from a number of measures including; Leiter-R Full Scale, Stanford Binet Full Scale, WISC-IV, WPPSI-III, Autism Spectrum Rating Scale, Autism Diagnostic Observation Schedule, Childhood Autism Rating Scale, Autism Diagnostic Interview, Mullen Early Learning Composite, Vineland-II Adaptive Behaviour Composite	ASD diagnosis in medical notes. Where known, the measure used was reported in Supplementary material	ASD: *n* = 25 (53%), ID: *n* = 15 (IQ < 80, average = 65), 18 more had diagnosis of ID or developmental delay with no scores. IQ range: 39–124, ASD and ID (*n* = 10), 16 participants (34%) had additional behavioural/ psychological diagnoses, including: learning disabilities, social communication disorder, disruptive behaviour disorder, ADHD, depression, bipolar disorder, OCD, ODD and/or aggression	0.5
Lachlan et al. (2009), UK	[9]	Individuals with known PTEN mutations were recruited through UK clinical genetics services	42	N/A	26 (M)	4–75 years	Search of molecular and histological reports and clinical details	N/A	Motor delays and learning difficulties. 12% (2/17) of non-probands had learning difficulties	0.58
*Lynch et al. (2009), Ireland	[46]	Review of genetic and neurology records between 2004 and 2007 for PTEN mutation. No referral information available	6	N/A	5 (M)	2 years 7 months–8 years at diagnosis	Unknown	ASD diagnosis in medical notes (no further detail)	Learning difficulties (*n* = 1), autistic features (*n* = 2), motor delay (*n* = 5), Asperger Syndrome (*n* = 1), language delay (*n* = 1) and speech delay (*n* = 2)	0.5
*Smpokou et al. (2014), USA	[47]	Electronic records of all patients seen at Boston Children’s hospital between 1996 and 2011 were searched for “PTEN”, “Bannayan-Riley-Ruvalcaba”, and “Cowden”. No referral information available	34	N/A	23 (M)	2–26 at last clinical evaluation	Developmental evaluation by a developmental paediatrician or clinical psychologist. Documentation of attainment of developmental milestones by either a clinical geneticist or a paediatric neurologist and records review	ASD classification based on clinical/researcher developmental appraisal	Developmental or intellectual disability, language delay, motor delay and ASD.	0.58
*Vanderver et al. (2014), International	[48]	Patients referred for unclassified white matter disorders who had clinical features of BRRS and abnormal PTEN sequencing or identified based on macrocephaly and/or developmental abnormalities with brain MRI and tested positive for PTEN mutation. In almost all cases, referrals were due to concerns related to macrocephaly and developmental delay	23	N/A	13 (M)	Newborn–5 years	MRI and review of clinical history	ASD diagnosis noted in clinical history (no further detail)	Developmental delay (*n* = 23), autistic features (*n* = 2), ASD (*n* = 5), motor delay (*n* = 4)	0.58
Yehia et al. (2019), International	[49]	Medical records of patients diagnosed with CS, CS-like and BRRS	511 (309 with confirmed PTEN)	N/A	161 (M)	1–89 years (mean = 45 years)	Review of medical records	N/A	ASD (*n* = 45), global developmental delay (*n* = 64), “mental retardation” (*n* = 12), learning disability (*n* = 10).	0.50
*Yehia et al. (2020), International	[50]	Participants were recruited from community and academic medical centres internationally between Sept 2005 and Jan 2018. Inclusion criteria included meeting relaxed Cowden syndrome diagnosis, macrocephaly plus a neurodevelopmental disorder and/or penile freckling or a known PTEN mutation. Checklist completed and blood specimen drawn along with medical records review	481	N/A	213 (M)	Mean = 33.2 SD = 21.6 years	Review of medical records	ASD diagnosis in medical records (no further detail)	ASD or developmental delay (*n* = 110), no evidence of ASD or DD (*n* = 194)	0.58

Note. *GAD* generalised anxiety disorder, *OCD* obsessive compulsive disorder, *ADHD* attention deficit hyperactivity disorder, *COWA* Controlled Oral Word Association Test, *AVLT* Auditory Verbal Learning Test, *ID* Intellectual delay, *ODD* oppositional defiant disorder, *ASD* autism spectrum disorder. *Included in meta-analysis

**Table 4:** Summary characteristics of group B articles

Author, year of publication, country of study	Reference Number	Recruitment procedure	PTEN sample size (total *n*)	Gender in PTEN patients	Age	Assessment tools/methods	Specific details of ASD definition/assessment (for papers used in meta-analysis)	Findings	Quality score
Butler et al. (2005), USA	[14]	Referrals to general genetics or autism clinics for diagnosis, medical management and/or genetic testing. For 6 participants, DNA was obtained from the Autism Genetic Resource Exchange and selected based on the diagnosis of classical autism and having macrocephaly	3 (18)	3 (M)	2 years 6 months–4 years	Autism Diagnostic Interview-Revised 5, clinical genetics evaluation. Psycho-behavioural examinations. Review of family and medical histories	N/A	Severe speech delay (*n* = 2), developmental delay (*n* = 2), speech apraxia (*n* = 1), short attention span (*n* = 2), language delay (*n* = 1) and ASD (*n* = 3)	0.89
Buxbaum et al. (2007), International	[12]	Recruited through the Paris Autism Research International Sibpair study at clinical centres internationally. Further recruitment by the Mount Sinai School of Medicine and/or the Autism Genetic Resource Exchange (AGRE). Participants with head circumference ≥ 2 SD were studied	5 (88)	3 (M)	3 years 6 months–26 years	Clinical evaluation following DSM-IV criteria for ASD, Autism Diagnostic Interview-Revised or the Asperger Syndrome Diagnostic Interview	N/A	Asperger Syndrome (*n* = 1), ASD (*n* = 4), developmental delay (*n* = 1), speech and language delay (*n* = 1) and delayed motor skills (*n* = 1)	0.89
*Kato et al. (2018), Japan	[51]	Genetic investigation of 33 Japanese patients with macrocephaly and development delay. No referral information given	6 (33)	2 (M)	4–6 years	Kinder Infant Development Scale (KIDS), Tanaka-Binet Intelligence Scale V, Kyoto Scale of Psychological Development	1 child described as having “autistic tendencies” with no further details provided	Developmental delay (*n* = 1), motor delay (*n* = 4), speech delay (*n* = 3), autistic tendencies (*n* = 1) Developmental Quotients; 76, 65, 85, 59, 54, 30	0.67
Klein et al. (2013), USA	[52]	Chart review of patients seen at UCLA genetics clinic from 2008 to 2011 with ASD and macrocephaly. Patients are referred to this clinic by a neurologist or a psychiatrist who had evaluated the patient using various autism screening and assessment measures	5 (33)	5 (M)	2 years 6 months–15 years	Autism Diagnostic Observation Schedule, Pre-Linguistic Autism Diagnostic Observation Schedule, Checklist for Autism in Toddlers, and Screening Tool for Autism in Toddlers & Young Children	N/A	ASD	0.78
*McBride et al. (2010), USA	[53]	Medical records searched of patients who have had PTEN clinical sequencing tests performed from January 1, 2008, to June 30, 2009, at a Children’s hospital.	4 (93)	1 (M)	8 months–9 years 4 months	Medical records review, with some reporting use of Autism Diagnostic Observation Schedule	Diagnoses made using DSM-IV criteria (ADOS used for confirmation in 20%)	Developmental delay (*n* = 2), ASD (*n* = 2), mental retardation (*n* = 1), affective disorder (*n* = 1), behavioural problems (oppositional and anger, *n* = 1)	0.67
Negishi et al. (2017), Japan	[54]	Unknown. All patients had increased head circumference and neurological symptoms (such as developmental delay and epilepsy)	3 (13)	0 (M)	4 years 2 months–4 years 9 months	Kinder Infant Development Scale (KIDS)	N/A	Developmental delay Developmental quotient = 59, 76 and 85	0.56
O’Roak et al. (2012), USA	[55]	Autistic probands recruited from Simons Simplex Collection. Probes used to target 44 ASD candidate genes	3 (2495)	2 (M)	Unknown	Unknown	N/A	ASD Non-verbal IQ = 50, 33, 77	0.56
*Orrico et al. (2009), Italy	[56]	Patients referred for genetic counselling due to macrocephaly associated with cognitive and behavioural impairment with or without features of ASD.	3 (40)	2 (M)	5–9 years	Vineland Adaptive Behaviour Scales and Childhood Autism Rating Scale (CARS)	Classification of ASD based on CARS	Moderately impaired communication, daily living skills, social interaction and motor skills (*n* = 1), severe ASD (*n* = 1), overall low adaptive behaviour and communication, daily living skills, socialisation and motor skills (*n* = 1)	0.78
*Saskin et al. (2017), USA	[57]	Analysis of whole-exome data from National Database for Autism Research.	6 (2392 families)	Unknown	Unknown	Unknown	ASD classification (further details unknown)	ASD (*n* = 2) and developmental delay (*n* = 1)	0.56
*Varga et al. (2009), USA	[58]	Search of medical records of a list of patients who had clinical PTEN gene sequencing ordered between January 1, 2005, and December 31, 2007, at a children’s hospital. Records were most commonly requested from molecular and human genetics and neurology and developmental disabilities/autism clinics.	11 (114)	8 (M)	3 months–35 years	Variety of autism assessments including Autism Diagnostic Observation Scale and search of medical records	Indicated in medical records based on DSM-IV criteria. Further information shows a range of professionals diagnosed ASD including developmental paediatricians, multidisciplinary evaluation, neurologists, psychiatrics or other physicians. ADOS used for confirmation for 13 participants	ASD (*n* = 5), developmental delay without ASD (*n* = 6) and expressive speech delay (*n* = 1)	0.67
*Wong et al. (2018), Hong Kong	[59]	Patients with suspected PHTS (indicated by autistic features and/or neurodevelopmental delays and macrocephaly) were referred for assessment and genetic testing to the Clinical Genetic Service (CGS) of Department of Health between January 1995 and September 2016. Records were also retrieved	3 (13)	2 (M)	9–10 years	Unknown	“Autism” or “Autistic features” stated in clinical features (further details on how this was diagnosed is not available)	Intellectual disability (*n* = 1), Autistic features (*n* = 3) and developmental delay (*n* = 2)	0.67
Yeung et al. (2017), Hong Kong	[60]	Patients recruited from January 2013 to December 2016 at the Duchess of Kent Children’s Hospital Child Assessment Center. Patients with ASD/DD and macrocephaly were assessed by a developmental paediatrician and allied health professionals. No further referral information	4 (21)	4 (M)	1 year 8 months–8 years 2 months	Griffiths Mental Developmental Scales-Extended Revised if less than 72 months, Hong Kong Wechsler Intelligence Scale if over 72 months, Autism Diagnostic Observation Schedule	N/A	Mild Global developmental delay (*n* = 3), moderate developmental delay (*n* = 1)	0.78

Note. *Included in meta-analysis

Overall, quality appraisal/risk of bias scores ranged from 0.5 to 0.89 (M = 0.64, SD = 0.12). Quality scores of the first author (KC) are presented in Tables 5 and 6. However, all studies were appraised independently by the second author (AWa), and inter-rater reliability was found to be excellent (two-way random effects, consistency, average-measures intraclass correlation coefficient for the overall score was .99, 95% CI .97–.99).

**Table 5:** Quality appraisal scores for Group A papers

Author	Sample Identification	Confirmation of syndrome	Symptom assessment	Comparison/control group	Total	Quality score
Busch et al (2013) [41]	1	3	3	1	8	0.67
Vanderver et al (2014) [48]	2	3	2	0	7	0.58
Smpokou et al (2014) [47]	1	3	3	0	7	0.58
Frazier et al (2015) [44]	0	3	3	2	8	0.67
Balci et al (2018) [39]	1	3	2	0	6	0.50
Busa et al (2015) [40]	1	3	2	0	6	0.50
Yehia et al (2020) [50]	2	3	2	0	7	0.58
Busch et al (2019) [42]	2	3	3	2	10	0.83
Ciaccio et al (2019) [43]	2	3	2	0	7	0.58
Hansen-Kiss et al (2017) [45]	1	3	2	0	6	0.50
Lachlan et al (2007) [9]	2	3	2	0	7	0.58
Lynch et al (2009) [46]	1	3	2	0	6	0.50
Yehia et al (2019) [49]	1	3	2	0	6	0.50

**Table 6:** Quality appraisal scores for Group B papers

Author	Sample Identification	Confirmation of syndrome	Symptom assessment	Total	Quality score
Orrico et al. (2009) [56]	1	3	3	7	0.78
Varga et al (2009) [58]	1	3	3	6	0.67
McBride et al (2010) [53]	1	3	2	6	0.67
O’Roak et al (2012) [55]	2	3	0	5	0.56
Klein et al (2013) [52]	1	3	3	7	0.78
Saskin et al (2017) [57]	2	3	0	5	0.56
Kato et al (2018) [51]	0	3	3	6	0.67
Yeung et al (2017) [60]	1	3	3	7	0.78
Negishi et al (2017) [54]	0	3	2	5	0.56
Butler et al (2005) [14]	2	3	3	8	0.89
Buxbaum et al (2007) [12]	2	3	3	8	0.89
Wong et al (2018) [59]	1	3	2	6	0.67

### Participant characteristics

#### Group a

The 13 studies in group A reported on a total of 1263 participants (51% male) with confirmed *PTEN* mutations or a *PTEN*-related condition (although, as with research into many rare syndrome groups, it is not possible to ascertain whether there is overlap between samples in some of these papers) and 93 control participants (including those with ASD with or without macrocephaly but no *PTEN* mutation and typically developing controls). Sample size ranged from six to 511 individuals. Age range varied from newborn to 89 years, with some studies reporting only a mean age. Only five studies included participants over the age of 30. The recruitment process was variable, and often only limited information about this was provided. Five papers had recruited people solely on the basis of *PTEN* mutations, three papers on the basis of *PTEN* mutations and some other feature (e.g. white matter lesions/disorders), and four papers reported on patients diagnosed with CS/BBRS and/or confirmed *PTEN* mutations.

Only two studies made comparisons of behavioural/psychological characteristics of individuals with *PTEN* mutations with other groups. Frazier et al. [44] used comparison groups of individuals with macrocephaly and ASD (*n* = 16), ASD without macrocephaly (*n* = 38) and healthy controls (*n* = 14). Busch et al. [42] included a comparison group of individuals with macrocephaly and ASD (*n* = 25).

#### Group B

Group B papers (*N* = 12) reported on a total of 5353 participants, including data from two large prevalence studies: O’Roak et al. and Saskin et al. [55, 57]. A total of 56 participants in these papers (1.0%) had a confirmed *PTEN* mutation or diagnosis of PHTS (confirmed number of cases ranged from three to 11 per paper), with age ranging from 1.6 to 35 years. Two studies did not provide demographic data specifically for those with *PTEN* mutations [55, 57]. The nature of the overall samples varied, with participants recruited for studies on the basis of ASD (3 studies), macrocephaly and ASD (2 studies), macrocephaly and other developmental/cognitive/behavioural/neurological symptoms (3 studies), suspected PHTS (1 study) or having been tested for PTHS/*PTEN* mutation (2 studies).

### Measures

In group A, four papers (31%) utilised or reported neuropsychological testing or measures, with seven (54%) gathering their data through medical records or developmental review and two (15%) not stating how the characteristics were assessed. In group B, nine papers (75%) reported at least one neuropsychological measure, and three studies did not provide this information [55, 57, 59].

The most commonly used measure to identify autistic features or record a diagnosis of ASD (where a measure was identified at all) was the Autism Diagnostic Observation Schedule (ADOS; [61]), used in five studies. The Autism Diagnostic Interview-Revised (ADI; [62]) was also frequently used (four studies). A range of measures were used to determine cognitive ability, including various editions of the Wechsler Adult Intelligence Scale [63] and the Wechsler Memory Scale [64].

### Common themes

A range of characteristics were noted in those with a diagnosed *PTEN* mutation (Table 7). Papers in group A reported on a wider range of difficulties, including emotional difficulties [39, 45] and specific types of cognitive impairments [41, 42, 44]. Group B largely focussed on the ability levels of their participants with only Orrico et al. [56] and McBride et al. [53] reporting emotional difficulties in their studies.

**Table 7:** Summary of the neurodevelopment, behavioural and cognitive characteristics reported in the papers included in the review

	Author	ASD/autistic features	Unspecified social communication disorder	Intellectual disability/low IQ	Unspecified developmental delay	Communication and speech/language delays	Motor delays or difficulties	Attention impairment/ADHD/ADD	Working memory	Memory impairment	Executive dysfunction
Group A	Busch et al. (2013) [41]			•			•			•	•
	Vanderver et al. (2014) [48]	•			•		•				
	Smpokou et al. (2014) [47]					•					
	Frazier et al. (2015) [44]			•					•		
	Balci et al. (2018) [39]	•		•		•	•	•		•	
	Busa et al. (2015) [40]	•				•	•				
	Yehia et al. (2020) [50]	•									
	Busch et al. (2019) [42]	•		•		•	•	•	•		•
	Ciaccio et al. (2019) [43]	•		•	•						
	Hansen-Kiss et al. (2017) [45]	•	•	•				•			
	Lachlan et al. (2007) [9]			•			•				
	Lynch et al. (2009) [46]	•				•	•				
	Yehia et al. (2019) [49]	•		•	•						
Group B	Orrico et al. (2009) [56]	•	•			•	•				
	Varga et al. (2009) [58]	•			•	•					
	McBride et al. (2010) [53]	•		•	•						
	O’Roak et al. (2012) [55]	•		•							
	Klein et al. (2013) [52]	•									
	Saskin et al. (2017) [57]	•			•						
	Kato et al. (2018) [51]	•			•	•	•				
	Yeung et al. (2017) [60]				•						
	Negishi et al. (2017) [54]				•						
	Butler et al. (2005) [14]	•			•	•	•	•			
	Buxbaum et al. (2007) [12]	•			•	•	•				
	Wong et al. 2018 [59]	•		•	•						

	Author	Reduced processing speed	Sensory dysfunction	Anxiety	Depression	Bipolar disorder	Obsessive compulsive disorder	Affective disorder	Aggression	ODD/disruptive behaviour disorder/problem behaviour	Psychotic episode	Poor adaptive functioning	Self-harm
Group A	Busch et al. (2013) [41]												
	Vanderver et al. (2014) [48]												
	Smpokou et al. (2014) [47]												
	Frazier et al. (2015) [44]	•										•	
	Balci et al. (2018) [39]	•		•		•	•				•		•
	Busa et al. (2015) [40]												
	Yehia et al. (2020) [50]												
	Busch et al. (2019) [42]	•	•							•		•	
	Ciaccio et al. (2019) [43]												
	Hansen-Kiss et al. (2017) [45]			•	•	•	•		•	•			
	Lachlan et al. (2007) [9]												
	Lynch et al. (2009) [46]												
	Yehia et al. (2019) [49]												
Group B	Orrico et al. (2009) [56]											•	
	Varga et al. (2009) [58]												
	McBride et al. (2010) [53]							•		•			
	O’Roak et al. (2012) [55]												
	Klein et al. (2013) [52]												
	Saskin et al. (2017) [57]												
	Kato et al. (2018) [51]												
	Yeung et al. (2017) [60]												
	Negishi et al. (2017) [54]												
	Butler et al. (2005) [14]		•										
	Buxbaum et al. (2007) [12]												
	Wong et al. 2018 [59]												

*Note.* • indicates the characteristic is noted to be present or reported at an elevated level

### ASD and autism spectrum characteristics

ASD or autistic features were the most frequently reported characteristic of participants and were reported in 19 studies (76%).

### ASD prevalence meta-analysis

**Fig. 2:**
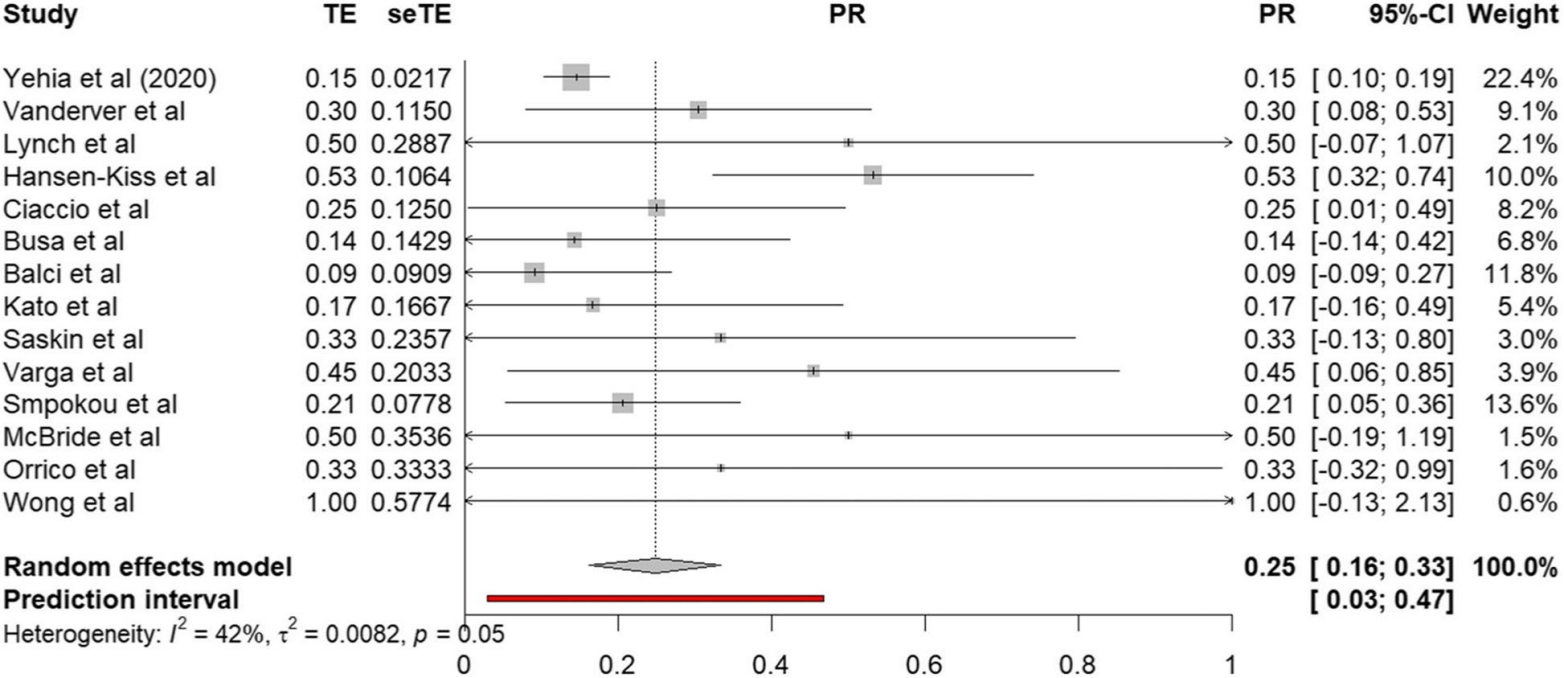
Forest plot of characteristics of ASD prevalence

Fourteen papers reported the prevalence of ASD or characteristics of ASD (or “autistic features”/ “autistic tendencies”; [54, 59]) in their participants with PTEN mutations or PHTS, with a total number of 486 participants, and prevalence ranging from 9 to 100%. Where the total number of participants and the authors were the same for two studies, it was deemed probable that the same participant group had been used in data analysis (Yehia et al. [49, 50]). In this case, the sample which was more specifically defined was used for the meta-analysis (Yehia et al. [50]). The random effects model (see Fig. 2) suggested a weighted average prevalence of 25% (95% CI 16–33%; *z* = 5.63, *p* < 0.001). An acceptable level of heterogeneity was observed (Higgin’s *I*^2^ = 42%; *τ*^2^ = 0.008, *Q*(13) = 23, *p* = 0.048).

The quality effects model gave a similar weighted average prevalence (24%, 95% CI 16–33%; *z* = 5.5, p < 0.001; *I*^2^ = 42%; *τ*^2^ = 0.008; Q (13) = 22, *p* = 0.048).

**Fig. 3:**
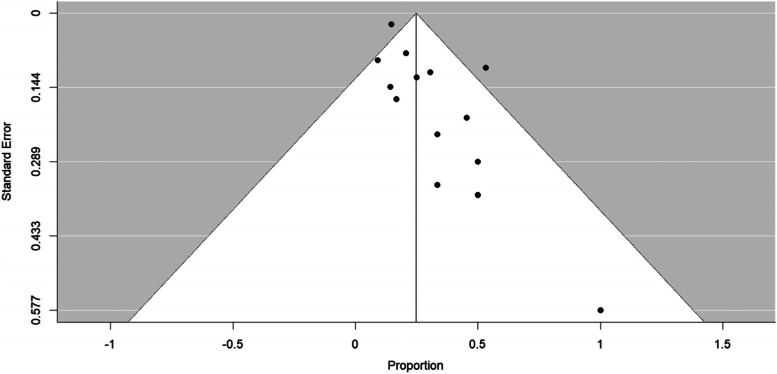
Funnel plot of characteristics of ASD prevalence. The 95% confidence interval of the expected distribution is shown as an inverted “funnel”

There was evidence of possible publication bias (Fig. 3), supported by a significant Egger’s test of funnel plot asymmetry (bias 1.13, *t* (12) = 3.17, *p* = 0.008). Using the trim and fill procedure [37, 38], six studies were introduced, leading to an imputed estimate of prevalence of 17% (95% CI 8–27%).

The meta-analysis was also re-run including only studies with 10 or more total participants, due to the tendency for studies with smaller sample sizes to show greater variability in their measurement. This also omitted the two papers [51, 59] which reported “autistic features” or “autistic tendencies” rather than ASD per se. This did not markedly affect prevalence estimates, with the random effects model again estimating a pooled prevalence of 25% (95% CI 14–36%). The meta-analysis was also re-run with group A papers only, since group B papers may be subject to extra/different sources of sampling bias (since samples comprised subsets of larger clinical groups, often defined by specific clinical characteristics). With only the eight papers from group A, the estimated prevalence was similar, at 23% (95% CI 13–33%).

### Other aspects of ASD

A number of studies suggested relationships between ASD and other characteristics for participants with *PTEN* mutations. Three studies [41, 42, 44], compared participants with both *PTEN* mutations and ASD with other groups. These data indicated that *PTEN* mutations, lower general functioning and ASD may be interrelated: participants with *PTEN* mutations and ASD were more greatly impaired in a number of domains, including overall intellectual functioning, attention, inhibition, expressive and receptive language and motor coordination, than those with *PTEN* mutation but no ASD [42], and participants with *PTEN* mutations and ASD had lower average ability (including lower processing speed (*d* = 1.15), working memory (*d* = 1.07), auditory immediate memory and adaptive function most notably community living) than individuals with ASD associated with macrocephaly without *PTEN* mutations [44]. Effect size was reduced following adjustment for IQ scores (processing speed: *Χ*^2^ = 3.71, *p* = 0.054 and working memory: *Χ*^2^ = 2.63, *p* = 0.105). Scores on the ADI-R did not significantly differ between those with ASD and a PTEN mutation and those with ASD with or without macrocephaly [44] suggesting that levels of ASD symptomatology for those with *PTEN* mutations may not differ significantly from levels of ASD symptomatology reported for those with ASD of different aetiology. Unfortunately, however, scores on ASD measures representing ASD severity were reported only infrequently and not in a manner allowing robust comparison other groups (e.g. McBride et al. [53] reported ADOS II score for a single participant only).

Sensory dysfunction, which features in DSM-5 criteria for ASD [65], was reported in one study [42]. Caregivers of participants with ASD and *PTEN* mutations observed greater symptoms of sensory dysfunction than the caregivers of those with *PTEN* mutations without ASD and the caregivers of children with macrocephaly and ASD. However, participants with *PTEN* mutations but without ASD were also reported to have impaired sensory processing. Butler et al. [14] also described sensory integration difficulties in one participant.

### Cognitive ability, developmental delay and intellectual disability

Participants’ ability levels were defined, assessed and reported in a variety of ways across papers. Some provided specific intelligence quotients (IQ), others report developmental quotients, and still others stated only whether intellectual disability or developmental delay was present for individuals.

IQ was reported in three studies in group A, and four studies in group B. Hansen-Kiss et al. [45] found that 15 of 47 participants (32%) identified as having a *PTEN* mutation, PHTS, CS or BRRS in their electronic records had an IQ of less than 80 (mean = 65), with 18 more (38%) having a documented history of intellectual disability or developmental delay. The full IQ range in this study was 39 to 124. This variation may relate to their recruitment method of searching medical records and therefore not limiting participation to those who can complete certain measures. Busch et al. [41] and Lachlan et al. [9] reported global impairments (borderline or lower IQ) or learning difficulties in 12% of participants, respectively. Busch et al. [41] reported IQs ranging from 80 to 120. It must be noted that participants were required to sit through four hours of neuropsychological testing, which may have resulted in individuals with lower not volunteering the study. O’Roak et al. [55] reported the non-verbal IQs of three participants (with their overall sample selected on the basis of having ASD), ranging from 50 to 77.

Other descriptions of cognitive ability/disability included Yehia et al. [49] reporting a learning disability or “mental retardation” in 22 (4%). In group B, intellectual disability was reported in three papers (e.g. in one of four [53] and one of three [59] participants). Some papers did not differentiate between intellectual disability and developmental delay [43, 47]. In these papers, intellectual disability and/or developmental delay were reported in 92% and 56% of participants respectively.

Developmental quotients (DQ) were also used to describe ability levels of participants in three group B studies [51, 54, 60], based on a variety of measures including the KIDS [66] and Kyoto Scale of Psychological Development [67]. DQs ranged from 30 [51] to 85 [51, 54] across these studies.

Developmental delay (often with no further specification) was reported in 48% of the reviewed studies (*n* = 12; three group A studies and nine group B papers). Reported prevalence in group A papers varied: Ciaccio et al. [43] and Vanderver et al. [48] reported 9 of 16 and 23 of 23 participants respectively to have a developmental delay, and Yehia et al. [49] reported global or variable developmental delay in 91 out of 511 participants. In group B, prevalence rates of developmental delay in participants ranged from 16 to 66% (excluding Yeung et al. [60] who looked at *PTEN* in this population).

Amongst specific developmental delays, motor delay was the most commonly reported (*n* = 11 studies, seven in group A). Busch et al. [41] found that participants with PHTS scored significantly lower than normative data in motor functioning (*t*(22) = − 5.02, *p* = .001, *d* = − .94), specifically in fine manual dexterity. Kato et al. [51] described motor delay in four of their six participants with a *PTEN* mutation. In this group, participants began walking between 14 and 29 months, with three participants walking after 26 months and showing motor delay. As well as delays in walking, general and psychomotor delays [56] and fine motor delays [14] were also reported.

Along with motor delays, speech and language delays were reported in five studies in group A and 5 studies in group B. Prevalence rates varied between 27 and 57% across the studies [39, 40, 46] in group A. Smpokou et al. [47] did not delineate between motor and language delays. Reports of profiles of ability across domains for individuals are rare. Busa et al. [40] report on one participant with an uneven ability profile, with scores within most indices on the WAIS in the “normal” range but a working memory index of 67.

### Attention, executive functioning and memory

Attentional difficulties were reported in four studies (three in group A). Reduced working memory abilities and processing speed were reported at group level in three group A studies [39, 42, 44]. Attention Deficit Hyperactivity Disorder (ADHD) was reported in two of 11 participants by Balci et al. [39], and in an unspecified number by Hansen-Kiss et al. [45], and a short attention span was noted in two of three individuals in one study [14].

Two studies in group A [39, 41] identified poorer memory functioning in their participants with *PTEN* mutations. Busch et al. [41] reported a difference between people with *PTEN* mutations and data from a normative comparison group in the memory recall domain (small effect size [*d* = 0.38]), with 12 participants (47%) showing reduced performance on a memory recall measure, although no significant differences were found in recognition memory. Balci et al. [39] reported two patients (18%) with memory problems indicated in medical records (with no further details).

Busch et al. [41, 42] reported impairments in the executive functioning domain, in which participants with *PTEN* mutations overall scored significantly lower than population controls (*d* = − 0.7, *p* = 0.001).

As previously mentioned, Frazier et al. [44] found that the large effect sizes for deficits in processing speed and working memory reduced following adjustment for full-scale IQ. However, when exploring the cognitive abilities of those with PHTS, most (88%) of whom had IQ scores in at least the low average range, Busch et al. [41] noted greater difficulty on measures of verbal fluency and fine motor skills than controls.

### Emotional difficulties

In group A, four studies reported emotional difficulties, including mental health diagnoses, in their participants. Balci et al. [39] reported two of 11 paediatric participants were diagnosed with generalised anxiety disorder. One participant had also received a diagnosis of obsessive-compulsive disorder (OCD) following a suicide attempt and self-harm. A psychotic episode was reported in a 5-year-old [39]. Hansen-Kiss et al. [45] reported diagnoses of disruptive behaviour disorder, oppositional defiance disorder, aggression, anxiety, depression, bipolar disorder and OCD in 34% (*n* = 16), but with no further delineations. “Problem behaviour” and poor adaptive functioning was more commonly reported in those with *PTEN* mutations and ASD than just *PTEN* mutations [42, 44].

Emotional difficulties were less frequently reported in group B studies. A nine-year-old female was reported to be diagnosed with an unspecified affective disorder and behavioural problems which were described as “oppositional and anger” with no further information [53].

## Discussion

The current review examines literature reporting psychological and behavioural characteristics in individuals with constitutional *PTEN* mutations. The 25 studies meeting criteria for the review fell into two categories: those that investigated the characteristics of individuals recruited after confirmed *PTEN* mutations or PHTS (group A), and those which assessed for presence of *PTEN* mutations in a sample of participants with specific characteristics, such as ASD and macrocephaly (group B). There was a similar number of studies in each group, although the total number of participants with *PTEN* mutations was considerably greater for group A.

ASD was the most commonly reported characteristic. A meta-analysis of the prevalence of ASD or characteristics of ASD (including 14 papers) revealed a weighted average prevalence of 25% (95% CI 16–33%). This was not markedly changed by weighting papers by risk of bias/quality ratings, by including only group A papers, by omitting papers with ten or fewer participants, or by omitting those who referred to “features of ASD” rather than ASD. Asymmetry of papers’ reported prevalence around the weighted average raised the possibility of publication bias; it is possible that ASD prevalence remains unreported/unpublished where this prevalence is lower. Following correction for possible publication bias, the estimate of prevalence decreased to 17% (95% CI 8–27%). However, even at the estimated lower confidence interval for this lower estimate, prevalence exceeds that in the general population (1–2% [68, 69];). The calculated prevalence adjusted for publication bias is similar to that estimated in neurofibromatosis type 1 (18%) and Down’s syndrome (16%), as noted by Richards et al. [19]. The impact of possible publication bias suggests that more large-scale studies looking at ASD and ASD characteristics in those with *PTEN* mutations and PHTS are needed to accurately estimate prevalence. Further to this, authors should endeavour to report prevalence of diagnoses in clinical samples where this information is available even if this is low.

Reviewed literature also suggests that individuals with ASD and *PTEN* mutations may differ on a number of psychological/behavioural dimensions from those without ASD, with evidence of lower ability in a number of areas (see below) for those also carrying diagnoses of ASD. This is in line with evidence that ASD is associated with lower ability more generally [70]. However, there was also evidence that those with *PTEN* mutations and ASD may also have more difficulties than those with ASD and macrocephaly of different aetiology [42], suggesting that the combination of *PTEN* mutations and ASD may be particularly associated with lower abilities, an association that should be explored more in further research.

There was evidence of overall reduced IQ for individuals with *PTEN* mutations. It should also be noted, however, that individuals’ reported cognitive abilities varied greatly, with some papers reporting on individuals with IQs over two standard deviations above the population mean (e.g. Busch et al. [41] reported an IQ range of 80–135). Data using standardised measures suggest that people with PTEN and ASD may on average have impairments in a number of domains [42, 44], including attention, working memory, processing speed, language, visual-spatial abilities. Where no ASD is present, the picture may be less clear, with one study indicating that a group of 23 individuals with PTEN mutations but no ASD did not score statistically significantly differently from normative comparison groups on some measures of cognitive functioning, including attention and processing speed [42]. However, given the low sample size, the possibility of a type 2 error due to low power must be considered. Despite associations between ASD and lower ability, the frequency with which ASD occurs in the absence of significant impairments in general ability remains a question for future research.

Where standardised measures of motor abilities were used at a group level, functioning was found to be significantly lower for people with BBRS/CS than in normative data [41] and was impaired for people with *PTEN* mutations both with and without ASD (although more so for those with ASD) [42]. However, again, there is apparent variability at the individual level.

Attention, executive functioning and memory were also reported to be impaired at a group level [39, 41, 42, 44, 45]. A small number of individuals were reported to have diagnoses of ADHD [39, 45], although this was not widely investigated/reported across papers so the degree to which impairments relate to specific developmental diagnoses is unclear. It has been postulated that deficits in processing speed and working memory associated with *PTEN* mutations may be related to poorly developed white matter [42, 44], and details of this possible association should be explored in further research. Full-scale IQ has been shown to be significantly related to executive functioning [71] and scores on tasks tapping into working memory contribute to a full-scale IQ score. For this reason, when exploring impairments in these domains, it is important to question whether these are to be expected given the individual’s IQ. The relative degree of impairment in these domains for people with PTEN differences, and the strength of relationships between impairments in different domains of cognitive functioning, are yet to be fully explored. Future research should build on existing work [41, 44].

Emotional difficulties were reported/assessed only sporadically. Where reported, there were suggestions that the prevalence of these difficulties may be high: Hansen-Kiss et al. [45] identified these issues in 34% of their participants, citing anxiety, bipolar disorder and OCD (although with few further details). “Disruptive” or “problem” behaviour was also reported in three papers. However, the lack of systematic investigation, using established measures and appropriate comparison groups, precludes knowledge of whether emotional difficulties occur differently from or at a higher rate than in the general population and/or other genetic neurodevelopmental syndrome groups. How this may relate to other difficulties such as ASD (known to be associated with anxiety, for example), also remains to be ascertained.

Relationships between different behavioural/psychological variables and specific genetic, physical or physiological characteristics were not generally explored in the reviewed papers. The precise relationship between different *PTEN* variants and psychological corollaries remains to be delineated. Recent research has begun to explore this, e.g. Yehia et al. [50] found in their sample of 309 individuals with PTEN mutations that those with ASD/DD had an overall increased burden of copy number variants).

### Strengths and limitations in the literature

For most studies, the presence of a *PTEN* mutation had been confirmed for all participants. Two studies [41, 49], however, included participants with diagnoses of CS and BRRS but without a *PTEN* mutation (*n* = 204). The precise nature of the mutations, and the additional difficulties also featured in inclusion criteria/recruitment processes for many studies, leads to potential differences in the nature of participant groups. These factors mean that interpretation of synthesised results, including meta-analytic estimates, should remain cautious. It may also be that some of the heterogeneity (e.g., in reported ability levels) between studies reflects differences in recruitment of samples which cannot be entirely characterised in the present analysis (e.g., because of limited information given in the papers). This requires thorough consideration in future research.

Nine of the 25 studies recruited participants from multiple centres or databases either nationally [9, 55, 57] or internationally [12, 48], which may enhance some aspects of generalisability. However, the data are largely from Western countries, and definitions and constructs of ASD and psychological distress may not relate to individuals in other cultures.

Small sample sizes may reflect the rarity (and relatively newly-identified) nature of the condition, which is likely to result in underpowered analyses. *PTEN* mutations are not routinely tested for and participants were frequently recruited through hospitals or clinics which may have led to a general bias in the literature, as this indicates that there was significant concern about the individual prior to genetic testing.

The use of clinical review as a main method for identifying behavioural characteristics in these studies also introduces possible biases [72].

### Clinical and research implications

Clinical evaluation and support of individuals with *PTEN-*related conditions should consider a wide range of possible corollaries, including ASD, cognitive and intellectual functioning, motor development and potentially, emotional difficulties.

Future research should employ a range of validated, standardised behavioural measures to allow a more comprehensive identification of the various domains associated with the behavioural phenotype of individuals with PTEN mutations (e.g. [73, 74]). This will also aid more extensive comparisons with other groups (including those representing typical development, idiopathic ASD and other genetic neurodevelopmental conditions), which should also be included within studies, since this is important in defining the behavioural phenotype specifically associated with a syndrome group [75–77]. As more individuals with constitutional *PTEN* mutations are identified world-wide, and relevant support groups and databases grow, this may also allow researchers to assess psychological and behavioural factors for a greater number of individuals who may not otherwise have come to attention of services. This may then allow for samples which may be less biased towards specific difficulties, enhancing understanding of psychological/behavioural correlates of *PTEN* changes more broadly.

## Conclusion

A systematic review of existing research with groups of people with constitutional *PTEN*-related conditions suggested a number of possible psychological/behavioural corollaries. Our meta-analysis estimated a prevalence of ASD or characteristics of ASD of approximately 25% (95% CI 16–33%), although it should be noted that this estimate may be inflated by publication bias, and should be interpreted with caution due to the varied nature of recruitment and basis on which ASD is determined. Further research is required on the qualitative nature of ASD phenomenology within this group. Research also indicates lower average cognitive abilities than in the general population, especially when ASD is also present, frequent reports of global developmental delay, motor and speech delay and cognitive impairment in those with *PTEN* mutations and PHTS. Wide variation in cognitive abilities is also noted. The relationship of psychological/behavioural variables with physiological or genetic factors remains relatively unexplored. Many studies are small scale, relying on retrospective reviews of medical records or unclear psychological assessment methods, and use of comparison groups was limited in available research. Future research, using detailed and well-established psychological assessment tools and appropriate comparison groups, may elucidate in greater depth the profile of possible characteristics associated with aberrations affecting *PTEN*.

## Supplementary Information

**Additional file 1.**

**Additional file 2.**

**Additional file 3.**

## Data Availability

Supporting data is available from the corresponding author upon reasonable request.

## Abbreviations

ADI: Autism Diagnostic Interview-Revised
ADOS: Autism Diagnostic Observation Schedule
BRRS: Bannayan-Riley-Ruvalcaba syndrome
CS: Cowden syndrome
IQ: Intelligence quotient
OCD: Obsessive compulsive disorder
PHTS: PTEN hamartoma tumour syndromes.
PRISMA-P: Preferred Reporting Items for Systematic Review and meta-analysis protocols
PTEN: *P*hosphatase and *ten*sin homologue
