# Supplementary material for: Behavioural and psychological features of PTEN mutations: a systematic review of the literature and meta-analysis of the prevalence of autism spectrum disorder characteristics
Source: J Neurodev Disord. 2022 Jan 4;14:1. doi: 10.1186/s11689-021-09406-w (PMC8903687; doi:10.1186/s11689-021-09406-w)
Supplement: Supplementary file 1 — Additional file 1. [file 11689_2021_9406_MOESM1_ESM.docx]

881 papers identified through database searching

(CINAHL=31, PsycINFO=128, SCOPUS=242, Web of Science=476) and via manual searches (*n*=4)

Records after duplicates removed (*n*=723)

Excluded duplicates

(*n*=158)

Records screened

(*n*=723)

Records excluded on basis of title or abstract

(n=625)

Full-text articles assessed for eligibility

(n=98)

Full-text articles excluded

(*n*=73)

Case studies

(*n*=22)

Low N

(*n*=3)

No behavioural characteristics reported

(*n*=10)

No focus on PTEN mutation

(*n*=19)

Non-human/molecular studies

(*n*=5)

Reviews

(*n*=14)

Studies included in review

(*n*=25)

**Identification**

**Screening**

**Eligibility**

**Included**
