# Supplementary figures and images for: Behavioural and psychological features of PTEN mutations: a systematic review of the literature and meta-analysis of the prevalence of autism spectrum disorder characteristics
Source: J Neurodev Disord. 2022 Jan 4;14:1. doi: 10.1186/s11689-021-09406-w (PMC8903687; doi:10.1186/s11689-021-09406-w)

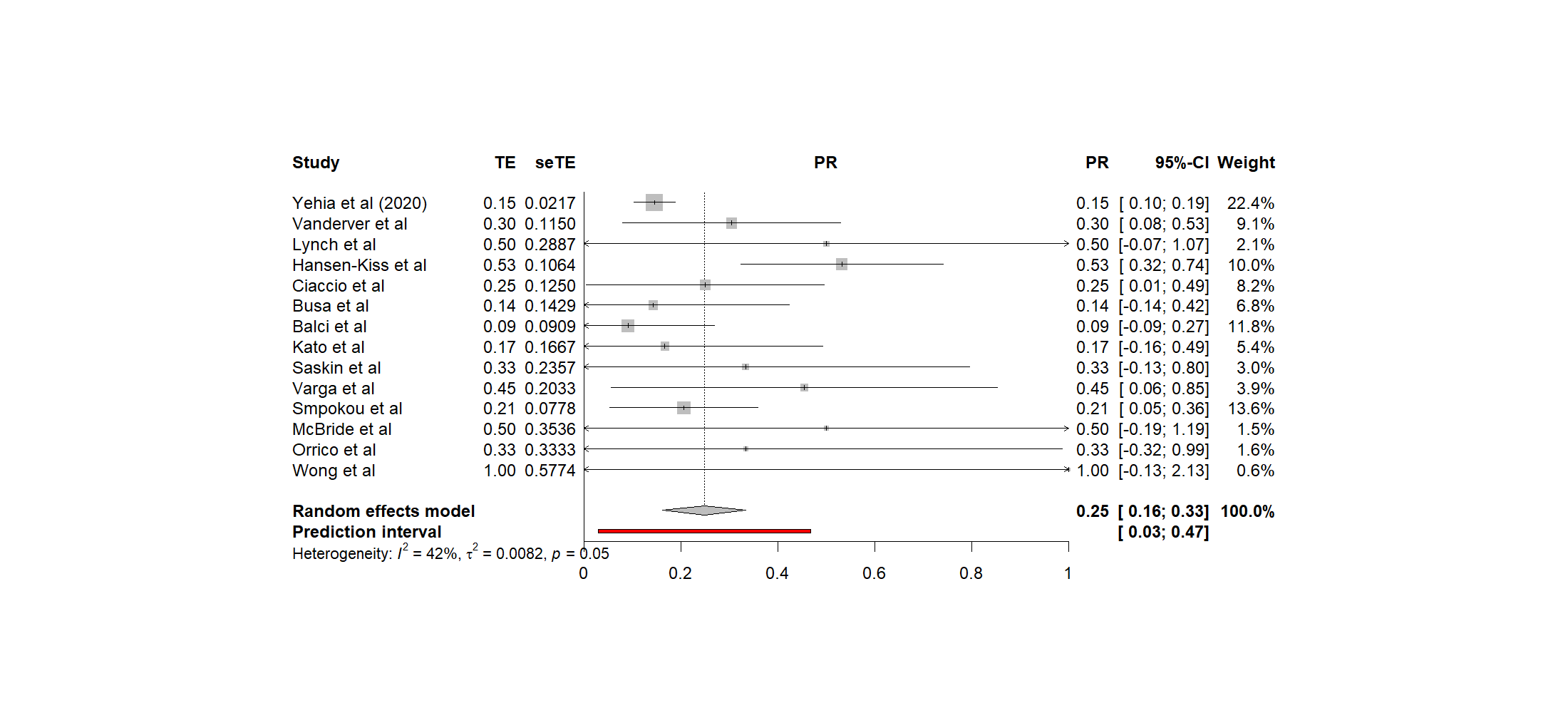

Supplement: Supplementary file 2 — Additional file 2. [file 11689_2021_9406_MOESM2_ESM.docx]

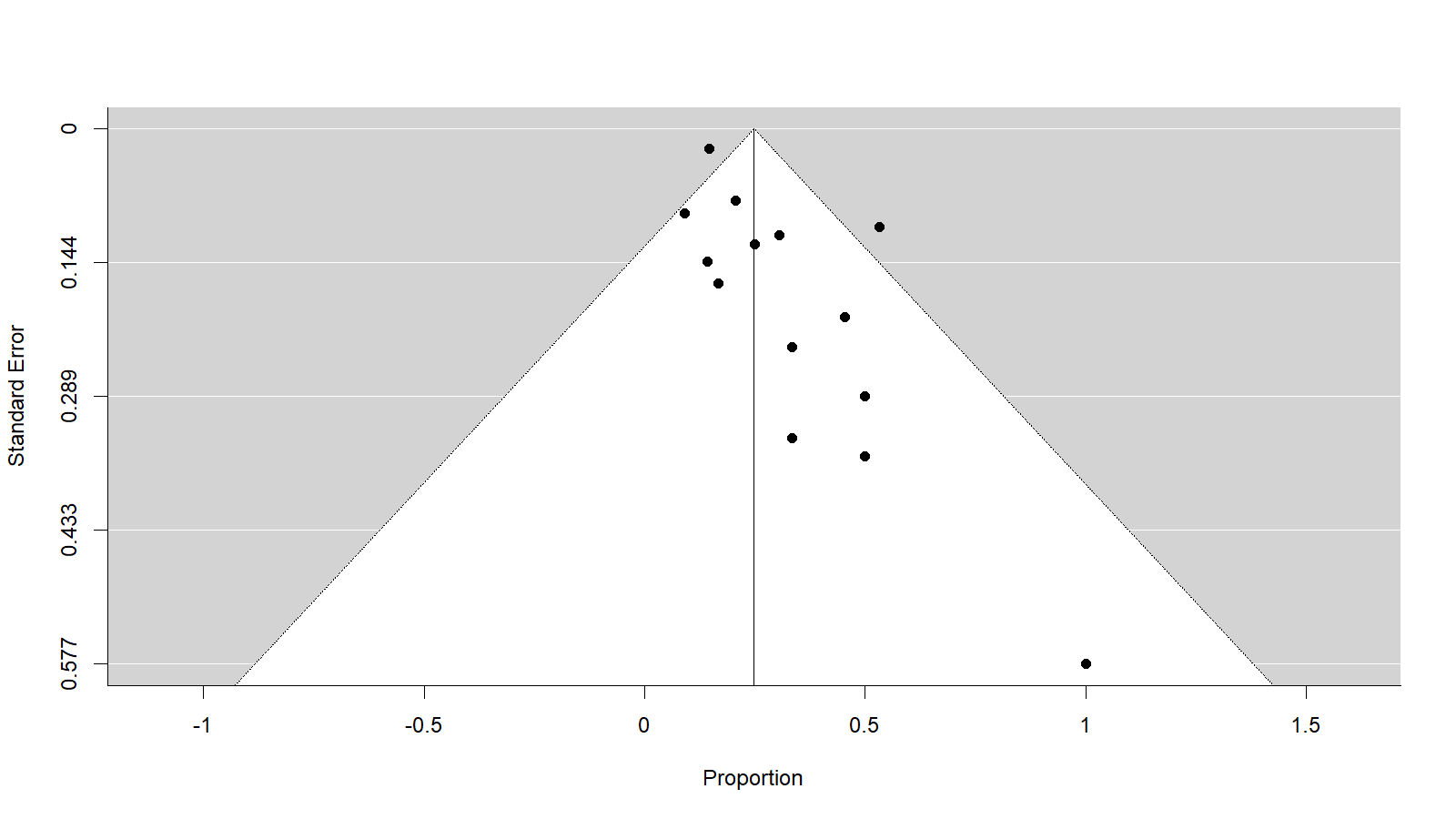

Supplement: Supplementary file 3 — Additional file 3. [file 11689_2021_9406_MOESM3_ESM.docx]
